# SARS-CoV-2 Nucleocapsid Protein is Associated With Lower Testosterone Levels: An Experimental Study

**DOI:** 10.3389/fphys.2022.867444

**Published:** 2022-06-03

**Authors:** Caio Henrique Lucio Carrasco, Paloma Noda, Ana Paula Barbosa, Everidiene Kinverlly Vieira Borges da Silva, Camila Gasque Bomfim, Bianca Helena Ventura Fernandes, Thiago Afonso Teixeira, Amaro Nunes Duarte Neto, Paulo Hilario Nascimento Saldiva, Kamal Achoa Filho, Cristiane Rodrigues Guzzo, Edison Luiz Durigon, Fernando Luiz Affonso Fonseca, Roseli Corazzini, Camilla Fanelli, Irene Lourdes Noronha, Jorge Hallak

**Affiliations:** ^1^ Androscience—Science and Innovation Center in Andrology and High-Complex Clinical and Research Andrology Laboratory, Sao Paulo, Brazil; ^2^ Division of Urology, Hospital Das Clinicas, University of Sao Paulo Medical School, Institute of Advanced Studies, University of Sao Paulo, Sao Paulo, Brazil; ^3^ Renal Division, Department of Clinical Medicine, Faculty of Medicine, University of São Paulo, São Paulo, Brazil; ^4^ Department of Microbiology, Institute of Biomedical Sciences, University of São Paulo, São Paulo, Brazil; ^5^ Reproductive Toxicology Unit, Department of Pathology, University of Sao Paulo Medical School, São Paulo, Brazil; ^6^ Institute of Advanced Studies, University of Sao Paulo, Sao Paulo, Brazil; ^7^ Department of Clinical Laboratory, University Center of ABC Medical School, Santo Andre, Brazil

**Keywords:** COVID-19, SARS-CoV-2, nucleocapsid, testis, testosterone, hypogonadism

## Abstract

The ongoing COVID-19 pandemic represents an extra burden in the majority of public and private health systems worldwide beyond the most pessimistic expectations, driving an urgent rush to develop effective vaccines and effective medical treatments against the SARS-CoV-2 pandemic. The Nucleocapsid structural viral protein is remarkably immunogenic and hugely expressed during infection. High IgG antibodies against Nucleocapsid protein (N protein) levels were detected in the serum of COVID-19 patients, confirming its pivotal antigen role for a T lymphocyte response in a vaccine microenvironment. Currently, adverse events associated with immunizations have raised some degree of concern, irrespective of its huge benefits in dealing with disease severity and decreasing mortality and morbidity. This hitherto study evaluates histological changes in rats’ testes, epididymis, prostate, and seminal vesicles and analyzes hormone levels after solely N protein inoculation. Therefore, we exposed a group of Lewis rats to weekly injections of the recombinant N protein for 28 days, while a control group was inoculated with a buffer solution. The N group revealed a more significant number of spermatozoa. Spermatozoa in the seminiferous tubules were counted in twenty 400 × microscopy fields (mean of 9.2 vs. 4.6 in the control group; *p* < 0,01), but significantly lower testosterone levels (mean of 125.70 ng/dl vs. 309,00 ng/dl in the control group; *p* < 0,05) were found. No other histological and biochemical changes were displayed. Conclusively, these data suggest testicular hormonal imbalance mediated by the SARS-CoV-2 N protein that could be linked to reported post-COVID-19 syndrome hypogonadism. More relevant research might be performed to confirm this viral antigen’s deleterious mechanism in the human testicular microenvironment, particular in Leydig cell function.

## Introduction

Coronavirus disease 2019 (COVID-19), caused by the severe acute respiratory syndrome coronavirus 2 (SARS-CoV-2), was declared a pandemic in March 2020 by the World Health Organization (WHO) (Gao et al., 2020), since then has already spread to more than 192 countries, causing an unprecedented collapse in the Public Health System with more than 365 million confirmed cases and more than 5.7 million confirmed deaths. (Kang et al., 2020; WHO, 2020). Cases range from mild to critical, 81 and 5% of the time, respectively. In many countries, the virus continues to spread rapidly, prompting a global effort in the race to develop new treatment modalities and vaccines to contain this global threat (Dutta et al., 2020; Aleem et al., 2022).

Thus, studies on SARS-CoV-2 revealed a genome composed of a single strand of messenger RNA, which encodes in its first two-thirds non-structural polyproteins and structural proteins in the final portion of the genetic material. Four of them are essential for viral replication; the two most prominent are called Spike protein (S protein) and Nucleocapsid protein (N protein), while Membrane proteins (M protein) and Envelope protein (E protein) are minor components. Currently, the S protein plays an essential role in the production of vaccines, but its high mutability and instability have imposed limitations on its use (Brian and Baric, 2005; Ramajayam et al., 2011; Wrapp et al., 2020). With the emergence of new variants, the need for new vaccines is a world emergency public health concern. In this context, the N protein emerged as a potential candidate due to its genomic stability and the homogeneity of amino acids in comparison to other betacoronaviruses that affect humans. It also demonstrated a high immune response, activating T lymphocytes and antibodies by infected patients (Leung et al., 2004; Okada et al., 2005; Grifoni et al., 2020; Oliveira et al., 2020; Aleem et al., 2022).

The role of SARS-CoV-2 infection in the male reproductive system, altering spermatogenesis and endocrine production with impaired fertility is well established. Multiple factors are involved such as the inflammatory cascade, direct cell damage and fever (Delli Muti et al., 2022), but little is known about the adverse effects of SARS-CoV-2 vaccines in a male reproductive tract, and clinical trials for this purpose are lacking. Current studies seek to understand the role of the vaccine in the male reproductive tract. Vaccines based on Spike protein were tested and showed no changes in seminal patterns such as concentration, motility, and morphology (Lifshitz et al., 2022). However, with the development of great number of vaccines, a greater understanding of the role of the various viral peptides in male fertility becomes necessary, testing their safety regarding spermatogenesis and the endocrine system, for example. (Yang et al., 2020).

Therefore, our objective in this study was to test the safety of a future vaccine based on N protein for male reproduction, analyzing the possible changes in testes, epididymis, seminal vesicles, and prostate gland of rats inoculated with the isolated N protein, as well as the impact of the effect of this immunizer on the serum levels of sex hormones such as total and free testosterone, luteinizing hormone (LH), and estradiol (E2). Such knowledge could determine the impact on male endocrine function, fertility and eugonadism or hypogonadism.

## Materials and Methods

### Nucleocapsid Protein Synthesis: Cloning, Expression, and Purification

To synthesize the N protein, SARS-CoV-2 RNA was isolated from the firsts Brazilian COVID-19 patients (GenBank: MT 350282.1) (Araujo et al., 2020), and reverse transcription was performed to obtain the virus Nucleocapsid cDNA, which was used to amplify the nucleocapsid DNA fragment by Polymerase Chain Reaction (PCR), using the primer sequences. The amplicon DNA fragment was purified using GeneJET PCR Purification kit (ThermoFisher Scientific) and digested with BamHI and NheI FastDigest enzymes (ThermoFisher Scientific). The digested amplicon DNA fragment was cloned into the expression vector pET-28a 2, previously digested with the same pair of restriction enzymes, using the T4 DNA Ligase enzyme (ThermoFisher Scientific). The DNA combination was allocated and used to transform chemically competent *E. coli* STELLAR cells (TaKaRa) and competent *E. coli* Stellar cells that were grown in 2xTY solid medium (16 g/L bacto-tryptone, 10 g/L yeast extract, 5 g/L sodium chloride, 1.5% agar) supplemented with kanamycin (50 μg/ml). A plasmid of positive clones was extracted using the GeneJET Plasmid Miniprep Kit following the manufacture protocol (ThermoFisher Scientific). Moreover, the nucleocapsid cloning was confirmed by digestion using BamHI and NheI FastDigest enzymes (ThermoFisher Scientific). The pET-28a containing the nucleocapsid DNA fragment was used to express the recombinant protein in the *E. coli* BL21 STAR (DE3) strain.

The differentiated cells were cultivated in a liquid 2xTY medium supplemented with kanamycin (50 μg/ml) and chloramphenicol (30 μg/ml). The differentiated cells were grown until OD600 nm of 0.6 was reached, under the agitation of 200 rpm at 37°C. At this stage, 0.5 mM isopropyl-β-D-1-thiogalactopyranoside (IPTG) was added to induce N protein expression for 4 h. The cells were harvested by centrifugation at 8,500 xg G for 15 min at 4°C. The pellet was resuspended in lysis buffer (50 mM MOPS pH 7.5, 200 mM sodium chloride, 5% (v/v) glycerol, 0.03% Triton-100 and 0.03% (v/v) Tween-20) and lysed by sonication in a Vibracell VCX750 Ultrasonic Cell Disruptor (Sonics, Newton CT) in an ice bath under agitation. The lysate was centrifuged at 30,000 xgG for 1 h at 4°C, and the supernatant was loaded to a HisTrap Chelating HP affinity column (GE Healthcare Life Sciences) previously equilibrated with 50 mM MOPS pH 7.0, 200 mM sodium chloride, and 20 mM imidazole buffer. Bound proteins were eluted over 20 column volumes using a 20–1,000 mM imidazole gradient. Samples of the eluted fractions were loaded into 15% SDS-PAGE gels, and the fractions containing the N protein were concentrated using Amicon Ultra-15 concentric filters (Merck Millipore) with a 10 kDa cutoff. The concentrated sample was loaded in a HiLoad 16/600 Superdex 75 pg column (GE Healthcare Life Sciences) for size exclusion chromatography previously equilibrated with 50 mM MOPS pH 7.0, 50 mM sodium chloride and 1 mM Ethylenediaminetetraacetic acid (EDTA) buffer, and the protein eluted in a 50 mM MOPS pH 7.0, 50 mM sodium chloride, and 1 mM EDTA buffer. Samples of the eluted fractions were loaded into 15% SDS-PAGE gels, and the fractions containing the N protein where the presence of the protein of interest in the solution was confirmed by Western Blotting (WB), and the fractions containing the protein were concentrated and stored at 4°C.

### Animals and Experimental Protocol

For the present study, we used twelve male albino Lewis rats, weighing between 300 and 400g, obtained from a colony established in the Central vivarium of the University of São Paulo Medical School (FMUSP). These animals were maintained at an ambient temperature of 23 ± 1°C, relative humidity of 60 ± 5%, and a 12/12 h light/dark cycle, with free access to conventional rodent chow and water throughout the study. All experimental procedures were approved by the Research Ethics Committee for the Use of Experimental Animals of the University of São Paulo Medical School (CEUA FMUSP Nº 1522/2020).

Seven Lewis rats were subjected to intramuscular (IM) injections of 100 µL of recombinant SARS-CoV-2 N protein diluted at 1,5 μg/μl in protein buffer, weekly, during four consecutive weeks (Lewis N protein). Additionally, following the same inoculation protocol, five Lewis rats received 100 µL of protein buffer (50 mM MOPS pH 7.0, 50 mM sodium chloride, and 1 mM EDTA and were used as control (Lewis Control). All animals had their body weight monitored weekly, and individual monitoring of diet (g) and water consumption (ml). By the end of the 4 weeks of study, the rats were subjected to isoflurane inhalation anesthesia and submitted to a laparotomy. Aortic blood and reproductive tissue were collected from the abdominal incision for hormonal measurements and histological analysis.

### Serum Hormonal Analyses

Blood was individually drawn from rats into tubes containing clot activator and centrifuged for 10 min, at 2,000 rpm, at 24°C. The serum was used for dosages of the following hormones: total and free testosterone, LH, and E_2_ were measured using a fully automated electrochem iluminescent methodology in Cobas 8000 Roche EQIA^®^ following good clinical analysis practices.

### Indirect Serum Biomarkers of COVID-19

Serum samples from the animals were also used to quantify C-reactive protein (CRP) and D-dimer, using specific immunoturbidimetric test kits (Wiener Lab, Rosario, Argentina), respectively. Finally, for Troponin I (TnL) dosage through reaction with specific antibodies and chemiluminescence reading, the kit (Wiener Lab, Rosario, Argentina) was used. All the analyses were performed in the SL 6000^®^ automatic analyzer (Wiener Lab Group).

### Histological Analysis

The reproductive tissue was dissected and fragmented in samples of; testes, seminal vesicles, epididymis, and prostate, which were dehydrated and paraffin-embedded through conventional techniques. The histological analysis of these samples was performed in 5-μm-thick sections and stained with hematoxylin-eosin.

For testes, epididymis, prostate, and seminal vesicles tissue evaluation, 20 consecutive microscopic fields of the male reproductive tract were observed under ×400 magnification. The presence of any thrombus, inflammation, epithelial atrophy, and histopathological abnormalities on these tissues were analyzed. In addition, damage to Leydig cells and the amount of sperm present in the seminiferous tubules were also observed for the testes. Seminiferous tubule cross-sections were counted in twenty 400 microscopy fields, and the number of spermatozoa per tubule cross-section was calculated, quantifying the spermatozoa in the interstice.

### Statistical Analyses

The results were analyzed by comparing groups, applying the Student’s *t*-test statistics, with “*p*” values lower than 0.05 being considered significant, to compare the results obtained in groups NP vs Control using the (GraphPad Prism^®^ software version 9.0). The results were presented as mean ± SE.

## Results

### Effects of Nucleocapsid Immunization on the Histology of the Male Reproductive Tract

**FIGURE 1:**
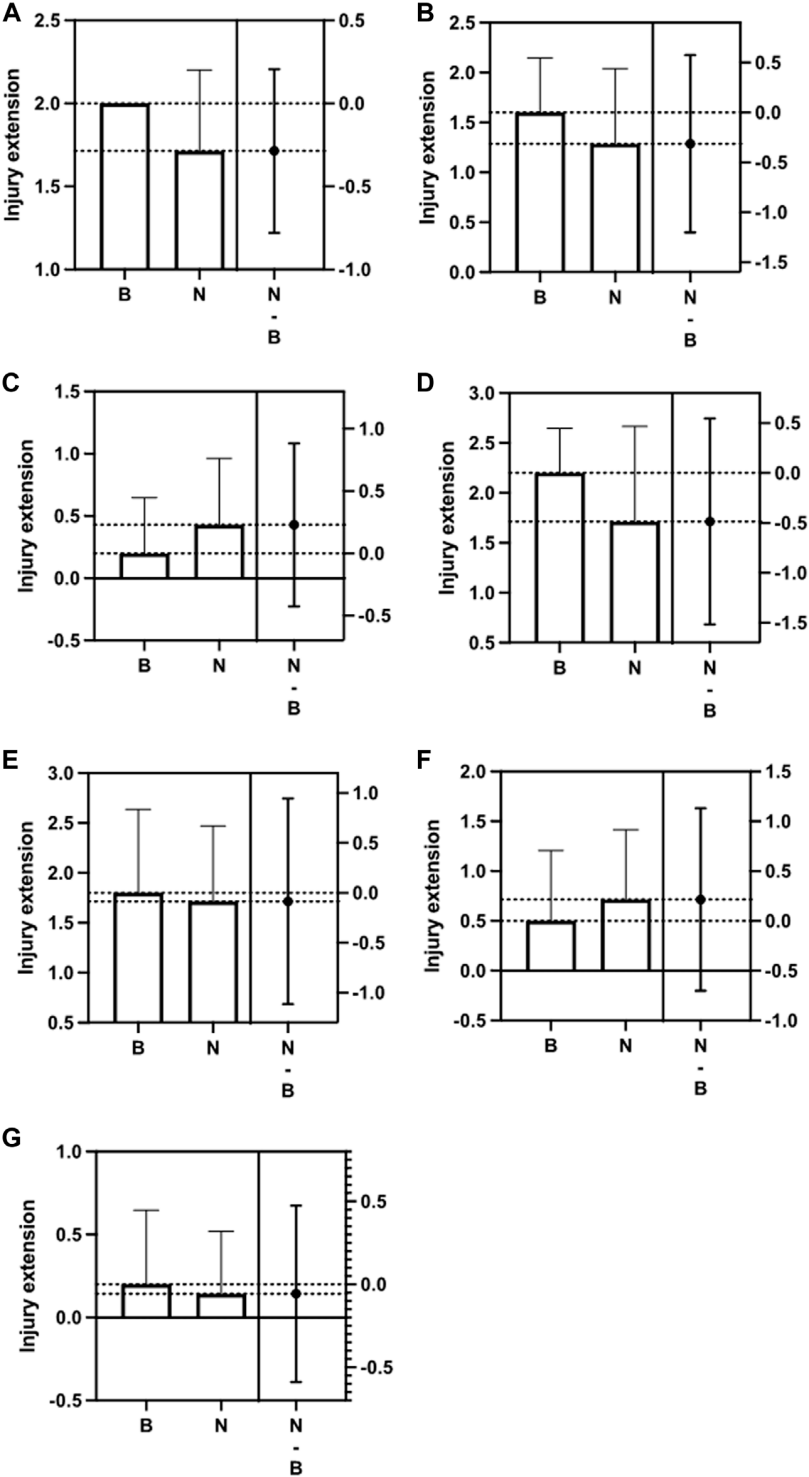
Difference between the Control group **(B)** and the Nucleocapsid group (N) in relation to Epididymis inflammation and epithelial atrophy **(A,B)**; Prostate inflammation and epithelial atrophy **(C,D)**; Seminal vesicle epithelial atrophy **(E)**; Testis inflammation and Leydig cell **(F,G)**; The second column indicates the difference between the means (*p* > 0.05).

Lewis rats were divided into two groups and inoculated weekly for 28 days with N protein, group N (*n* = 7), and with buffer solution, control group (*n* = 5). Table1. At the end of the period, we evaluated histopathological changes in the epididymis, prostate, seminal vesicles, and testes. For the analysis of the epididymis, prostates, and seminal vesicles, we considered the presence of thrombi, inflammation, and epithelial atrophy. We classified inflammatory changes and epithelial atrophy as none, mild, moderate, or severe according to T-cell infiltration or the extent of epithelial involvement, respectively. None of the groups presented thrombi in the structures studied. Comparing the epididymis, both groups demonstrated an inflammatory process and epithelial atrophy, ranging from mild to moderate, with no statistical difference between them (inflammation: *p* > 0.05; epithelial atrophy, *p* > 0.05). Figures 1A,B. About the prostate, the groups had minor inflammation. All of them had a mild appearance and the presence of moderate epithelial atrophy, with no significant variation between them (inflammation: *p* > 0.05; epithelial atrophy: *p* > 0.05). Figures 1C,D . No inflammatory process was observed in the seminal vesicles of these groups. Epithelium atrophy was present in both without disparities (*p* > 0.05). Figure 1E.

**TABLE 1:** Histopathological characteristics of epididymis, prostate, testis, and seminal vesicles.

	Epididymi			Prostate			Testis				Seminal vesicle		
*Lewis—Buffer Solution*													
Rat	Thrombus	Inflammation	Epithelial atrophy	Thrombus	Inflammation	Epithelial atrophy	Thrombus	Inflammation	Number of Spermatozoa	Leydig Cells	Thrombus	Inflammation	Epithelial Atrophy
*R1*	0	2	1	0	0	2	0	1	1	0	0	0	2
*R2*	0	2	2	0	0	2	0	1,5	5	0	0	0	1
*R3*	0	2	2	0	1	2	0	0	4	1	0	0	2
*R4*	0	2	1	0	0	3	0	0	5	0	0	0	3
*R5*	0	2	2	0	0	2	0	0	8	0	0	0	1
*Lewis—Nucleocapsid*													
*R6*	0	1	0	0	0	1	0	1	15	0	0	0	2
*R7*	0	2	1	0	0	3	0	0	7	1	0	0	1
*R8*	0	2	2	0	0	2	0	1	9	0	0	0	2
*R9*	0	2	1	0	0	1	0	1,5	9	0	0	0	1
*R10*	0	2	2	0	1	3	0	1,5	8	0	0	0	1
*R11*	0	2	2	0	1	1	0	0	11	0	0	0	2
*R12*	0	1	1	0	1	1	0	0	6	0	0	0	3

Classification according to the Thrombus: absence = 0, presence = 1; Inflamation and Epithelial atrophy injury extent: none = 0, mild = 1, moderate = 2, and severe = 3; Number of spermatozoa: counted in 204,00 × microscopy fields

**FIGURE 2:**
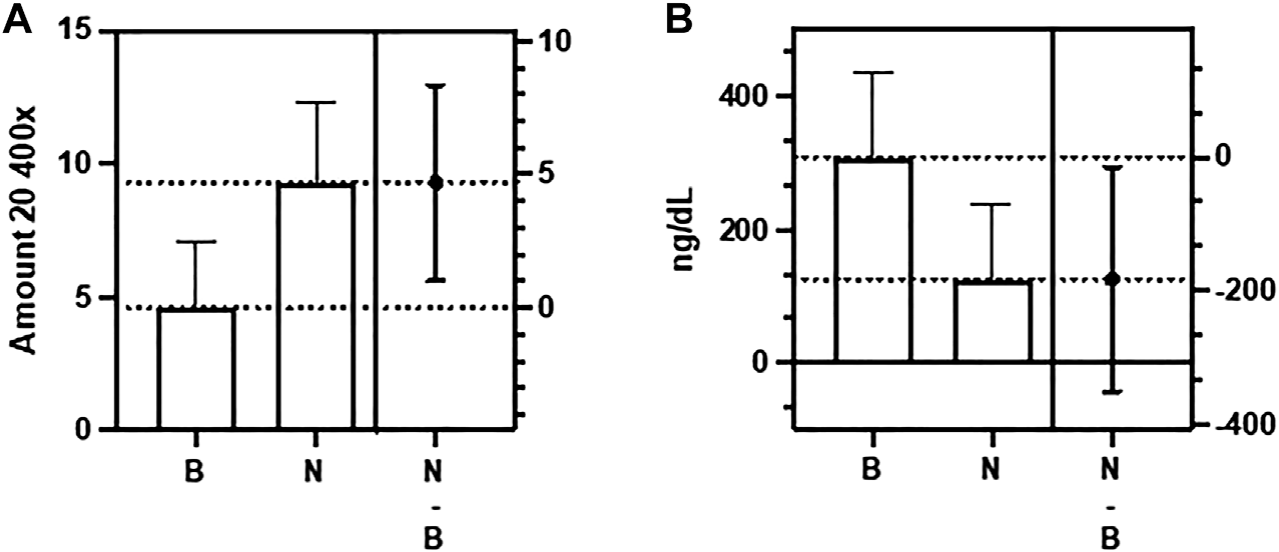
Difference between the Control group **(B)** and the Nucleocapsid group (N) in relation to number of spermatozoa in testis **(A)** and total testosterona **(B)** (*p* < 0,05).

In evaluating the testes, we considered, in addition to thrombi and inflammation, the number of spermatozoa present in the seminiferous tubules and the Leydig cell injury’s degree, also classified according to the extent of the damage. Regarding inflammation, it was practically not found in both groups (*p* > 0.05). Figure 1F Regarding the presence of sperm, we found a significantly higher amount in the exposed group, with a mean of 9.2 (range 6—15) compared to the control group (4.6; range 1–8; *p* < 0.05). Figure 2A. However, similar damage to Leydig cells was observed in both groups. Figure 1G.

### Serum Dosage of Male Sex Hormones in Rats Exposed to Nucleocapsid

To evaluate the effects of N protein inoculation on the serum levels of sex hormones, we used the same groups, the N group (*n* = 7) and the control group (*n* = 4). Table 2. Serum from one rat in the control group had to be discarded because it was not stored appropriately. At the end of the exposure period, a serum analysis of each animal was performed. For this, we quantified total testosterone (T) in ng/dl, free testosterone (FT) in ng/dl, LH in Mul/ml, and E2 in pg/ml. Table 3 presents the values of testosterones and its significant diference between the groups.

**TABLE 2:** Dosage of sex hormones.

Buffer group	E_2_	LH	T	FT
R2	9,6	0.3	484	20,2
R3	9,31	0.319	315	13,1
R4	9,31	0.327	180	7,48
R5	7,48	0.328	257	10,7
Nucleocapsid Group				
R6	6,93	0.309	57,5	2,38
R7	12,6	0.316	64,9	2,68
R8	15,1	0.31	171	7,1
R9	11,6	0.321	113	4,66
R10	10,8	0.326	14,2	0.58
R11	8,61	0.315	358	14,9
R12	8,7	0.319	101	4,18

E2, Estradiol (pg/ml); LH, luteinizing hormone (MuI/ml); T, total testosterone (ng/dl); FT, free testosterone (ng/dl).

**TABLE 3:** Total testosterone and free testosterone levels.

	Buffer Group	Nucleocapsid Group
Testosterone		
*Mean* ± *SD*	309 ± 129,1	125,7 ± 113,7
*Range* (*95% IC*)	103,6 to 514,4	20,50 to 230,8
Free testosterone		
*Mean* ± *SD*	12,87 ± 5,402	5,211 ± 4,738
*Range* (*95% IC*)	4,274 to 21,47	08,297 to 9,593

Student *t* test; *p* < 0,05.

Testosterone (ng/dl); Free testosterone (ng/dl).

There was no significant difference between the groups regarding the serum levels of LH (*p* > 0.05), and E2 (*p* > 0.05). However, we found a significant decrease in T levels in the N group, with a mean of 125.7 ng/dl (range 14.2—358 ng/dl) compared to the control group (mean of 309 ng/dl, range 180—484 ng/dl; *p* < 0.05) (Table 2). Decreased FT levels were also found in the Nucleocapsid group (mean 5.21 ng/dl, range 0.58—14.9 ng/dl) compared to the buffer group (mean 12.87 ng/dl). dL, range 7.48—20.2 ng/dl; *p* < 0.05) (Table 2).

## Discussion

This study aimed to evaluate the effects of the immune response with the inoculation of N protein on the histology of the male reproductive tract and the levels of sex hormones. Regarding histology, our results show a significant difference in the number of spermatozoa found between the groups, with a smaller amount present in the control group. However, we did not find significant histopathological changes between them, particularly in the seminiferous tubules. It is noteworthy that the groups did not present significant damage to the Leydig cells, responsible for intratesticular testosterone production, overall steroids synthesis and cellular-cellular communication with Sertoli cells to provide support for spermatogenesis and homeostasis of the testicular microenvironment (Zirkin and Papadopoulos, 2018). A study performed Doppler ultrasound on the testicles of 26 patients of reproductive age with COVID-19 sought to evaluate signs of orchitis that could corroborate microscopic lesions, but no changes were found (Carneiro et al., 2021). Other studies found lesions in the same cells in patients with COVID-19 but could not relate these changes to SARS-CoV-2 infection or an exacerbated immune response, much less were related to the N protein. The presence of the virus in testicular tissues could not be confirmed, nor could seminal alterations (Yang et al., 2020; Hallak et al., 2021). The role of the blood-testis barrier in protecting against the host’s immune response, preventing histopathological alterations, is questioned (Zhao et al., 2014; Pan et al., 2020a). As we only used N protein epitopes, we can exclude viral aggression as the cause of any finding. We are not aware of any publication that has addressed this issue before. Therefore, we conclude that immunization does not affect the male reproductive tract tissues.

Regarding the dosage of sex hormones, our data point to low serum levels of T and FT in the N protein group, with no statistically significant change in the other hormones. We know that Sertoli and Leydig cells play a fundamental role in producing of androgens and in the regulation of spermatogenesis, respectively (Griswold, 1998; Zirkin and Papadopoulos, 2018). Previous studies had already identified alterations in testicular endocrine function in 119 men of reproductive age infected with SARS-CoV-2, which had demonstrated an increase in LH levels and a decrease in the testosterone/LH ratio, probably reflecting an injury to Leydig cells, causing an early stage of transient hypogonadism (Ma et al., 2020; Drevet et al., 2021; Teixeira et al., 2021). In other reports, low testosterone levels were also identified in patients with COVID-19, but the samples showed alterations such as a reduction in Leydig cells, intense orchitis, and injury to the seminiferous tubules, which could explain the pathologic findings (Campos et al., 2021; Selvaraj et al., 2021; Duarte Neto et al., 2022). Hormonal regulation is more complex and dependent on sites and signals other than the gonads, which make up only a part of the hypothalamic-pituitary axis (Liu et al., 2018). Therefore, to better understand and interpret our results, further research should investigate the male endocrine system.

This study has some limitations. Our results may have been impacted by the small sample size, and more comprehensive immunohistochemical analyzes of discovered histopathological tissue changes were not performed. In addition, we used the control group animals inoculated with a protein solution, also found in the production of the N protein vaccine, which can generate a confounding factor during analysis. Another bias to be considered was the prolonged storage time of samples, both tissue and animal serum, in which preservation factors might have some degree of influence. We could not establish a relationship between low testosterone levels and changes in spermatozoa count, as this is the first study in the literature to address these alterations, other studies in the future should check for these variables. Considering that there was no measurement of sex hormones prior to inoculation, attention should be taken when interpreting the results of the influence of N protein on male endocrine function. However, our work was innovative in showing the effects of the host’s immune response to the inoculation of the N protein on the tissues of the male reproductive tract, elucidating that immunization has no role in reducing the number of spermatozoa and that the potential tendency to hypogonadism deserves attention, as it has been reported in numerous publications that address the post-COVID-19 syndrome (Drevet et al., 2021). Our findings suggest that prospective cohort studies with a more significant number of cases are necessary to determine whether the hormonal alteration has another influencing factor in addition to the host response.

This study describes the inoculation in rats of the recombinant protein Nucleocapsid, which has excellent potential as a vaccine. We found no evidence of significant tissue damage in testes, epididymis, prostate, or seminal vesicles. Hormones such as LH, and E2 revealed no changes, suggesting only testicular involvement by a mechanism not yet understood. However, our data should serve as guidance for further research to understand the impact of long-term immunizations on male reproductive function, including fertility, testicular endocrine function and male hypogonadism in the post-covid-19 Era.

## Data Availability

The datasets presented in this study can be found in online repositories. The names of the repository/repositories and accession number(s) can be found in the article/Supplementary Material.
